# Ponticelli regimen and nephrotic syndrome

**DOI:** 10.4103/0971-4065.57114

**Published:** 2009-07

**Authors:** S. Laxminarayana

**Affiliations:** Department of Nephrology, Amrita Institute of Medical Sciences, Kochi, Kerala, India

Sir,

We read the article of Das *et al*.[1] with interest; it raised few questions that beg answers.

Both in children and adults with steroid resistant minimal change disease (MCD) and focal segmental glomerulo sclerosis (FSGS), cyclosporine is the preferred choice.[2] Cytotoxic drug (cyclophosphamide) is indicated in frequent relapsing and/ or steroid dependent/toxicity group of MCD pediatric patients. The article was a mixture of frequent relapsers and steroid resistant nephrotic syndrome (SRNS). It is possible that the patients who improved with this protocol were only frequent relapsers, not affected with SRNS. Presuming that only cyclophosphamide (cytotoxic drug) is sufficient, addition of high dose of steroid adds to the steroid toxicity. The addition of prednisone to the cytotoxic drug may not improve the efficacy of the regimen, but it allows the patient for high fluid intake to protect against the cystitis. In such a case, the dose of steroid is tapered once remission is achieved.[3]

The authors were not explicit about the use of the terms, ‘frequent relapsers’ and ‘steroid resistant’ in the context of membranoproliferative glomerulonephritis and IgA nephropathy. IgA nephropathy is treated when proteinuria >1 g/24 h with preserved renal function with steroid for six months and with progressive renal failure (serum creatinine <2.84 mg/dl), a cytotoxic agent is added. The treatment in the latter group is at least for two years.[4] It is surprising that the lone IgA nephropathy with FR (sic) and renal impairment achieved remission and improvement in renal function at the end of six months. Results in fact did not present clinical and laboratory features of each group of the disease.

With expanding armamentarium to treat nephrotic syndromes, based on it etiology, proteinuria, renal impairment, renal biopsy features and individual merit, the authors surprisingly presented a unifying concept of a common treatment to all nephrotic syndromes, irrespective of its etiology. Is there a message, that the renal biopsy is, now, redundant; treat all with steroids, but if there is no response, use this protocol.[1]

The definition used for ‘renal failure’ in the article was persistent doubling of plasma creatinine over baseline value but the highest serum creatinine, mentioned only in Table 4, was only 1.12 + 0.58 mg/dl. In Table 3, another term ‘renal insufficiency’ was used, but it was not defined in the article.

## References

[1] Das U, Dakshinamurthy KV, Prasad N (2009). Ponticelli regimen in idiopathic nephrotic syndrome. Indian J Nephrol.

[2] Cattran DC, Alexopoulos E, Heering P, Hoyer PF, Johnston A, Meyrier A (2007). Cycloporin in idiopathic glomerular disease associated with nephrotic syndrome: workshop recommendations. Kidney Int.

[3] Dember LM, Salant DJ, Wilcox CS (2008). Minimal change disease. Therapy in Nephrology and Hypertension. A companion to Brenner and rector's The Kidney.

[4] Floege J (2003). Evidence-based recommendations for immuno-suppression in IgA nephropathy: handle with caution. Nephrol Dial Transplant.

